# A heuristic approach to determine an appropriate number of topics in topic modeling

**DOI:** 10.1186/1471-2105-16-S13-S8

**Published:** 2015-09-25

**Authors:** Weizhong Zhao, James J Chen, Roger Perkins, Zhichao Liu, Weigong Ge, Yijun Ding, Wen Zou

**Affiliations:** 1 Division of Bioinformatics and Biostatistics, National Center for Toxicological Research, U.S. Food and Drug Administration, Jefferson, AR 72079, USA; 2 College of Information Engineering, Xiangtan University, Xiangtan, Hunan Province, China

**Keywords:** Rate of perplexity change (RPC), perplexity, topic number, latent Dirichlet allocation (LDA)

## Abstract

**Background:**

Topic modelling is an active research field in machine learning. While mainly used to build models from unstructured textual data, it offers an effective means of data mining where samples represent documents, and different biological endpoints or omics data represent words. Latent Dirichlet Allocation (LDA) is the most commonly used topic modelling method across a wide number of technical fields. However, model development can be arduous and tedious, and requires burdensome and systematic sensitivity studies in order to find the best set of model parameters. Often, time-consuming subjective evaluations are needed to compare models. Currently, research has yielded no easy way to choose the proper number of topics in a model beyond a major iterative approach.

**Methods and results:**

Based on analysis of variation of statistical perplexity during topic modelling, a heuristic approach is proposed in this study to estimate the most appropriate number of topics. Specifically, the rate of perplexity change (RPC) as a function of numbers of topics is proposed as a suitable selector. We test the stability and effectiveness of the proposed method for three markedly different types of grounded-truth datasets: *Salmonella *next generation sequencing, pharmacological side effects, and textual abstracts on computational biology and bioinformatics (TCBB) from PubMed.

**Conclusion:**

The proposed RPC-based method is demonstrated to choose the best number of topics in three numerical experiments of widely different data types, and for databases of very different sizes. The work required was markedly less arduous than if full systematic sensitivity studies had been carried out with number of topics as a parameter. We understand that additional investigation is needed to substantiate the method's theoretical basis, and to establish its generalizability in terms of dataset characteristics.

## Background

Topic models are Bayesian statistical models where unstructured data, normally a set of textual documents, are structured in accordance with latent themes called topics that have multinomial distributions on words. Given a collection of unstructured text documents, topic modeling assumes that there are a certain number of latent topics in the collection of documents (corpus) and that each document contains multiple topics in different proportions. Researchers have developed several topic models, including Latent Semantic Indexing (LSA) [1], Probabilistic Latent Semantic Analysis (PLSA) [2,3], and Latent Dirichlet Allocation (LDA) [4]. Topic modeling has wide applications in various fields such as text mining [2-5], image retrieval [6], social network analysis [7] and bioinformatics analysis [8-11].

LDA, an unsupervised generative probabilistic method for modeling a corpus, is the most commonly used topic modeling method. LDA assumes that each document can be represented as a probabilistic distribution over latent topics, and that topic distribution in all documents share a common Dirichlet prior. Each latent topic in the LDA model is also represented as a probabilistic distribution over words and the word distributions of topics share a common Dirichlet prior as well. Given a corpus *D *consisting of *M *documents, with document *d *having *N_d _*words (*d *∈{1,..., *M*}), LDA models *D *according to the following generative process [4]:

(a) Select a multinomial distribution *φ_t _*for topic *t *(*t *∈{1,..., *T*}) from a Dirichlet distribution with parameter *β*.

(b) Select a multinomial distribution *θ_d _*for document *d *(*d *∈{1,..., *M*}) from a Dirichlet distribution with parameter *α*.

(c) For a word *w_n _*(*n *∈{1,..., *N_d _*}) in document *d*,

(i) Select a topic *z_n _*from *θ_d_*.

(ii) Select a word *w_n _*from *φ_zn_*.

In above generative process, words in documents are the only observed variables while others are latent variables (*φ *and *θ*) and hyper parameters (*α *and *β*). In order to infer the latent variables and hyper parameters, the probability of observed data *D *is computed and maximized as follows:

(1) $$p\left(D|\alpha ,\beta \right)=\overset{M}{\underset{d=1}{\prod }}\int p\left(\theta_{d}|\alpha \right)\left(\overset{N_{d}}{\underset{n=1}{\sum }}p\left(z_{dn}|\theta_{d}\right)p\left(w_{d_{n}}|z_{dn},\varphi \right)P\left(\varphi |\beta \right)\right)d\theta_{d}d\varphi$$

Due to the coupling between *θ *and *φ *in the integrand in Eq. (1), exact inference in LDA is intractable. Various approximate algorithms such as variational inference [4,6-8] or Markov chain Monte Carlo (MCMC) [5,9,11] are typically used for inference in LDA.

The effectiveness of LDA to segregate document collections into germane themes has been well demonstrated for document collections such as manually curated scientific literature where the "truth" within documents and the number of relevant themes are known *a priori *[10]; such sets of already structured documents are hereafter called truth sets. Difficulty arises, however, for unstructured document sets where document-wise content and number of relevant themes are not known *a priori*. That is, the best number of topics to enable the best topic model is unknown, while different numbers of topics will likely result in very different structuring of the corpus. An insufficient number of topics could render an LDA model that is too coarse to identify accurate classifiers. On the other hand, an excessive number of topics could result in a model that is too complex, making interpretation and subjective validation difficult [10]. We have been unable to identify any current efforts to develop a heuristic from which to evaluate an appropriate number of topics for a previously unseen and modelled, unstructured document set. Lacking such a heuristic to choose the number of topics, researchers have no recourse beyond an informed guess or time-consuming trial and error evaluation. For trial and error evaluation, an iterative approach is typical based on presenting different models with different numbers of topics, normally developed using cross-validation on held-out document sets, and selecting the number of topics for which the model is least perplexed by the test sets. Perplexity is a commonly used measurement in information theory to evaluate how well a statistical model describes a dataset, with lower perplexity denoting a better probabilistic model. Formally, for a test set of *M *documents, the perplexity is defined as $perplexity\left(D_{test}\right)=\text{exp}\left\{-\frac{\sum_{d=1}^{M}\text{log}p\left(w_{d}\right)}{\sum_{d=1}^{M}N_{d}}\right\}$[4]. Using the identified appropriate number of topics, LDA is performed on the whole dataset to obtain the topics for the corpus. We refer to this as the perplexity-based method.

Although the perplexity-based method may generate meaningful results in some cases, it is not stable and the results vary with the selected seeds even for the same dataset. In this study, we propose a new approach in which the rate of perplexity change (RPC) is calculated, and the change point of RPC is determined to be the most appropriate number of topics. The proposed approach is designated as RPC-based change point method (RPC is used hereafter). Three different types of datasets were applied to test the approach and the results validated the stability and effectiveness of the proposed method for selecting the best number of topics for LDA algorithms. The novel method was found to be unique, accurate, easy to use, and applicable to various kinds of datasets with different data types, and therefore, improving the accuracy and efficacy of topic model-based text mining and data mining.

## Materials and methods

### Datasets

In this study, three different types of datasets were utilized to test and evaluate the proposed approach. The first dataset is the whole genome sequences of 119 *Salmonella enterica *strains. The 119 *Salmonella *strains belong to *Salmonella *O antigen group B [12], including 75 *S*. Agona, 14 *S*. Heidelberg, 1 *S*. Paratyphi B, 2 *S*. Saintpaul, 2 *S*. Schwarzengrund, 1 *S*. Stanley, 22 *S*. Typhimurium, 1 *S*. Typhimurium var.5- and 1 *S*. 4,[5],12:i.

The second dataset was retrieved from the publicly available SIDER2 database (**http://sideeffects.embl.de**) [13]. The dataset includes 996 drugs with 4500 side effects filtered by MedDRA (Medical Dictionary for Regulatory Activities: http://www.meddra.org). The original dataset was represented by a 996 × 4500 drug-side effect matrix, where each entry is either 1 or 0, indicating presence or absence in the drug profile. In data preprocessing, each drug was considered as a document and each existing side effect term in a document was considered as a word in the vocabulary. The Anatomical Therapeutic Chemical (ATC) classification system (http://www.who.int/classifications/atcddd/en**/**) was applied to classify the 996 drugs in SIDER2 dataset according to their target organs or systems and their therapeutic, pharmacological and chemical properties. The ATC terms were utilized to evaluate the proposed method by calculating the *k*-means cluster purities.

We created the third dataset by retrieving the abstracts of papers published in the IEEE Transactions on Computational Biology and Bioinformatics (TCBB) from the PubMed database. The dataset was comprised of all the abstracts of 885 papers published in TCBB from 2004 to 2013. The dataset was preprocessed by tokenizing, removing stop words and stemming.

### Developing the heuristic approach to determine the appropriate topic number

Models were built using *m*-fold cross validation. Data were randomly divided into *m *subsets denoted as *S*_1_, *S*_2_, ..., *S_m_*. Candidate numbers of topics *t*_1_, *t*_2_,..., *t_r _*were sorted in increasing order. For each number of topics *t*, an LDA model was built *m *times on a training set combining *m*-1 subsets of the entire dataset. The trained LDA model was then utilized to calculate the perplexity on the held-out testing subset. Thus, each subset *S_i _*(*i*∈{1,...,*m*}) was included in the training set (*m*-1) times and tested once. The average of perplexities from *m *testing sets was taken to be perplexity result for each candidate number of topics. Denoting the average perplexities for *r *candidate number of topics as *P*_1_, *P*_2_... *P_r_*, the rate of perplexity change (RPC) for topic number *t*_*i *_(1<*i*≤*r) *was calculated as in Eq. (2).

(2) $$RPC\left(i\right)=|\frac{P_{i}-P_{i-1}}{t_{i}-t_{i-1}}|$$

The LDA algorithm implemented in Mallet [14] was used in our study, where inference in Mallet was based on Gibbs sampling [5].

### Method evaluation

#### Evaluation of method stability

The whole genome sequence dataset of 119 *S. enterica *strains was used to evaluate the stability of the proposed RPC-based change point method. The dataset was preprocessed and aligned with the multiple sequence alignment (MSA) algorithm MUSCLE [15]. Nucleotide differences among the sequences of 119 strains were taken to be single nucleotide polymorphisms (SNPs). Each resultant SNP and its corresponding coordinate location in the aligned sequence were encoded as a word.

To evaluate stability, the RPC-based method was compared with the perplexity-based method. The testing topic numbers were selected as 5, 10, and then increments of 10 more up to 100. Model building using cross-validation was repeated 50 times. Each time, a different random seed in Gibbs sampling from Mallet's program was used for each approach, and generated an appropriate topic number for each of the two methods. The frequencies of the obtained appropriate topic numbers were counted, and could be viewed as a probabilistic distribution over tested topic numbers after normalization. Then the entropy of the distribution was calculated to evaluate the stability of the two methods [16]. In information theory, entropy is a measurement to evaluate the uncertainty of a source of information. The Shannon entropy [16] was calculated as in Eq. (3).

(3) $$entropy\left(P\right)=\overset{t}{\underset{i=1}{\sum }}-P_{i}\cdot log_{2}P_{i}$$

where the distribution *P *is the normalized frequency of the derived appropriate topic numbers obtained in each of the approaches. The smaller the entropy value, the more stable the method.

#### Evaluation of method efficiency

Cluster analysis was conducted on the output of LDA models with various numbers of topics to evaluate the efficiency of the proposed method. For the sequence dataset of 119 *Salmonella *strains, leave-one-out (119-fold) cross validation was applied to calculate RPC values on the tested topic numbers 5, 10, plus increments of 10 up to 100. Hierarchical clustering algorithm and *k*-means algorithm with 10 clusters were conducted on the probabilities of obtained topics for all 119 strains. The purities of the resultant clusters were calculated based on the true labels (real serotypes of the strains). The average purities were considered as the final evaluation values for the LDA models with different number of topics. The running time of LDA models with different number of topics was compared to show the efficiencies of the proposed method.

Five-fold cross validation was applied on the SIDER2 dataset. Clustering analysis using the topic probabilities of the different drugs (documents) was conducted to comparatively evaluate the LDA models with different number of topics. Hierarchical clustering algorithm and *k*-means algorithm with two different settings of *k *(i.e., number of clusters) were used. Each cluster was labelled as the dominant ATC code among the drugs in the cluster and the ratio of the ATC code was calculated as purity of the cluster. The average purity of the obtained clusters by *k*-means method was used to evaluate LDA models with different numbers of topics. The running time of LDA models with different number of topics was also compared.

Five-fold cross validation was utilized to select the most appropriate number of topics for the TCBB dataset for the proposed method. The topic numbers 5, 10, and increments of ten up to 100 were used. A visual representation, word cloud, was created based on the distribution over words, and manually interpreted to evaluate the accuracies of the proposed method [17]. The word cloud generator (http://www.jasondavies.com/wordcloud) was used.

## Results

### Development of RPC-based method

**Figure 1:**
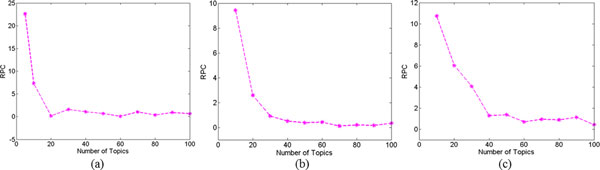
**RPC values of LDA models with various testing topic numbers in each of three datasets**. (a) *Salmonella *sequence dataset; (b) SIDER2 dataset; (c) TCBB dataset.

The RPC-based heuristic approach to select an appropriate number of topics for an LDA topic model was applied to three distinctly different datasets with very different data types. After data preprocessing as described in Material and Methods, the *Salmonella *sequence dataset was transformed into a corpus of 119 documents (corresponding to strains), where each document consisted of the same number of words (i.e., the number of SNPs after MSA). The final corpus had a total of 99,960 occurrences (119x840) in 119 documents that contained 2379 various SNPs. The SIDER2 corpus had a total of 117,329 occurrences in 996 documents and contained 4500 various words (i.e., side effects). The TCBB dataset corpus had a total of 84,646 occurrences in 885 documents (abstracts), and contained 5004 various words. RPC values for the LDA models at the candidate numbers of topics were calculated with *m*-fold cross validation for each of the three preprocessed datasets using Eq. (2). The results are plotted in Figure 1(a)-(c). Based on our method, the number of topics corresponding to the change of slope for the plot of RPC versus number of topics was deemed to be the most appropriate for a given dataset. That is, the first *i *that satisfied *RPC*(*i*) <*RPC*(*i*+1) was chosen as the most appropriate number. According the results in Figure 1, the best number of topics were 20, 50, and 40 for the *Salmonella *sequence dataset, SIDER2 dataset, and the TCBB dataset, respectively.

### Evaluation of the proposed RPC-based method

Three different datasets were used in this study to evaluate the stability and efficiency of the approach proposed to choose a best number of topics in LDA topic modelling.

#### Comparison of method stabilities

**Figure 2:**
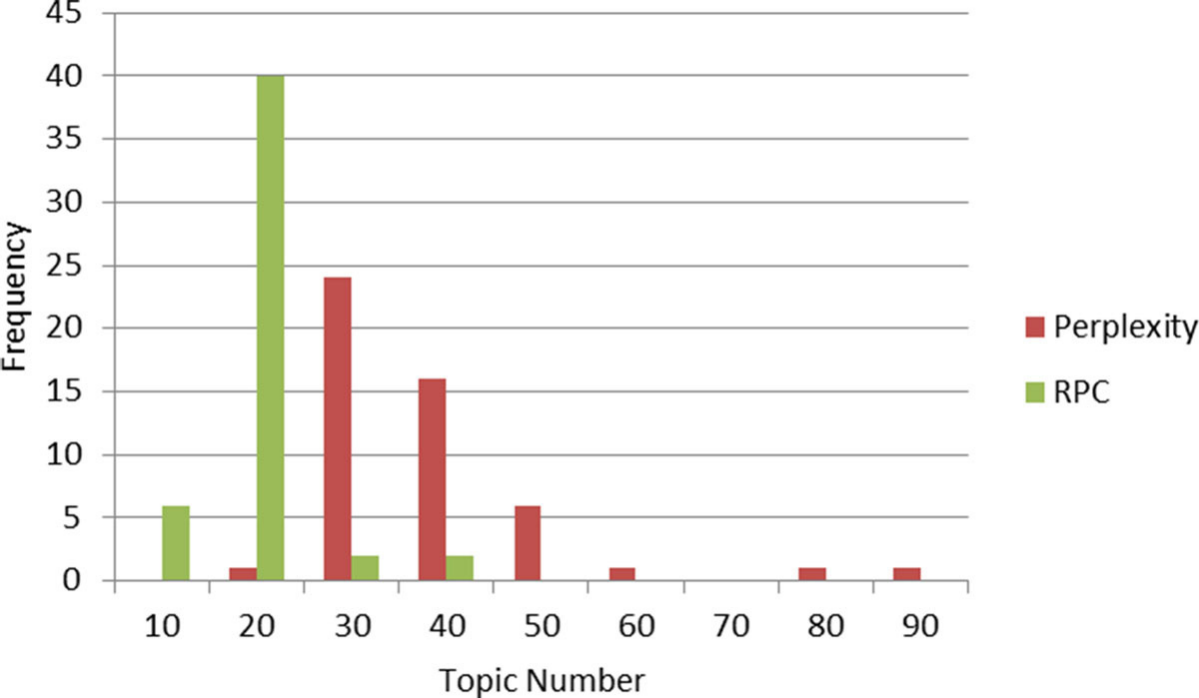
**Comparison of frequencies of candidate topic numbers obtained by perplexity-based method and RPC-based method**.

Both the perplexity-based approach (Perplexity) and the proposed RPC-based approach (RPC) were repeated 50 times with different random seeds to the *Salmonella *sequence dataset. Figure 2 plots the frequencies of the calculated most appropriate number of topics. The RPC-based method (green bars) chose 20 topics as most appropriate for 80% of the models, and 10, 30 or 40 topics for the remaining 20%. In contrast, the perplexity-based approach (red bars) appropriate ranged widely from 20 to 90 topics also, while 30 was selected as often most frequently, it was less in only 23 of 50 iterations. Additionally, the mean model entropy for the RPC-based method was 1.0, much lower than the 1.853 for perplexity-based models, further confirming RPC-based selection of numbers of topics to be the more stable approach.

#### Comparison of method efficiencies

LDA models were built for each of the three datasets for different numbers of topics, and of course including the selected appropriate numbers of topics. Each model's result provided matrices of topics, topic probability distributions across documents, and the word probability distributions across topics. The efficiencies of the proposed method were evaluated by data mining towards the derived LDA matrices from the three datasets.

Both hierarchical clustering and *k*-means clustering were performed on the *Salmonella *strains-topics (i.e, document-topic) LDA probability matrix for the *Salmonella *sequence dataset. The real serotypes of 119 *Salmonella *strains were used as the true labels to identify the misclassified strains. The resultant hierarchical cluster dendrogram trees for all numbers of topics considered yielded the highest purity when trees were cut at a height of 0.25. The numbers of misclassified strains from each hierarchical cluster and the LDA computing time for different number of topics are shown in Table 1. The results of *k*-means (*k *= 10) showed that LDA models with 20 or 30 topics gave the best clustering accuracy with all 119 strains correctly identified (Table 2). Since LDA models normally require more running time to converge with an increasing number of topics, 20 was determined as the most appropriate number of topics for the *Salmonella *sequence dataset based on both accuracy and efficiency. This result accommodates with that obtained by the proposed RPC-based approach.

**Table 1:** Hierarchical clustering accuracy and running time of *Salmonella *sequence dataset

T*	5	10	20	30	40	50
Misclassified	3	3	**0**	0	15	15
Time(ms)	33,914	34,584	**34,824**	35,478	35,636	35,816

***T***	**60**	**70**	**80**	**90**	**100**	

Misclassified	15	15	15	15	15	
Time(ms)	36,143	36,365	36,517	36,636	36,969	

*T: Number of topics.

**Table 2:** *K*-means clustering accuracy and running time of *Salmonella *sequence dataset

T	5	10	20	30	40	50
Purity** (*k *= 10)	0.95	0.93	**0.96**	0.96	0.93	0.93
Time(ms)	33,914	34,584	**34,824**	35,478	35,636	35,816

***T***	**60**	**70**	**80**	**90**	**100**	

Purity (*k *= 10)	0.93	0.93	0.93	0.93	0.93	
Time(ms)	36,143	36,365	36,517	36,636	36,969	

**Purity of each cluster is calculated as the ratio of correctly classified strains in the total 119 strains in the cluster. The ratios in the table represent the average purities of *k *clusters obtained for each topic modeling.

Hierarchical clustering and *k*-means algorithm with two different settings (*k *= 20 and 30) were also both utilized to cluster the drug-topic matrix derived from LDA models for the SIDER2 preprocessed dataset across the different numbers of topics. The 996 drugs in SIDER2 dataset were classified into 14 main groups according to the first level term of the ATC. To evaluate the accuracies of the proposed approach, the misclassified drugs from hierarchical clustering analysis and the purities of the *k*-means clusters were calculated on the basis of the ATC codes and classifications of the drugs as described in Material and Methods. The resultant hierarchical cluster dendrogram trees cut at a height of 0.6 showed that the least number of drugs (205) as misclassified when the number of topics was 50 (Table 3). Similar results shown in Table 4 confirms that the highest purities were obtained when the number of topics was 50 or 60. Because of the lower run time, 50 topics were considered as the most efficient.

**Table 3:** Hierarchical clustering accuracy and running time on SIDER2 dataset

T*	5	10	20	30	40	50
Misclassified	443	411	362	355	285	**205**
Time (ms)	43,378	45,233	48,252	49,278	50,493	**51,443**

***T***	**60**	**70**	**80**	**90**	**100**	

Misclassified	223	246	251	269	269	
Time (ms)	52,526	52,577	54,298	54,468	54,608	

*T: Number of topics.

**Table 4:** *K*-means clustering accuracy and running time of SIDER2 dataset

T	5	10	20	30	40	50
Purity**(*k *= 20)	0.41	0.44	0.53	0.53	0.53	0.58
Purity(*k *= 30)	0.41	0.44	0.56	0.50	0.54	**0.60**
Time (ms)	43,378	45,233	48,252	49,278	50,493	**51,443**

***T***	**60**	**70**	**80**	**90**	**100**	

Purity (*k *= 20)	**0.59**	0.55	0.57	0.56	0.54	
Purity(*k *= 30)	0.59	0.57	0.57	0.56	0.56	
Time (ms)	52,526	52,577	54,298	54,468	54,608	

**Purity of each cluster is calculated as the ratio of correctly classified drugs in the total 996 drugs in the cluster. The ratios in the table represent the average purities of *k *clusters obtained for each topic modeling.

**Figure 3:**
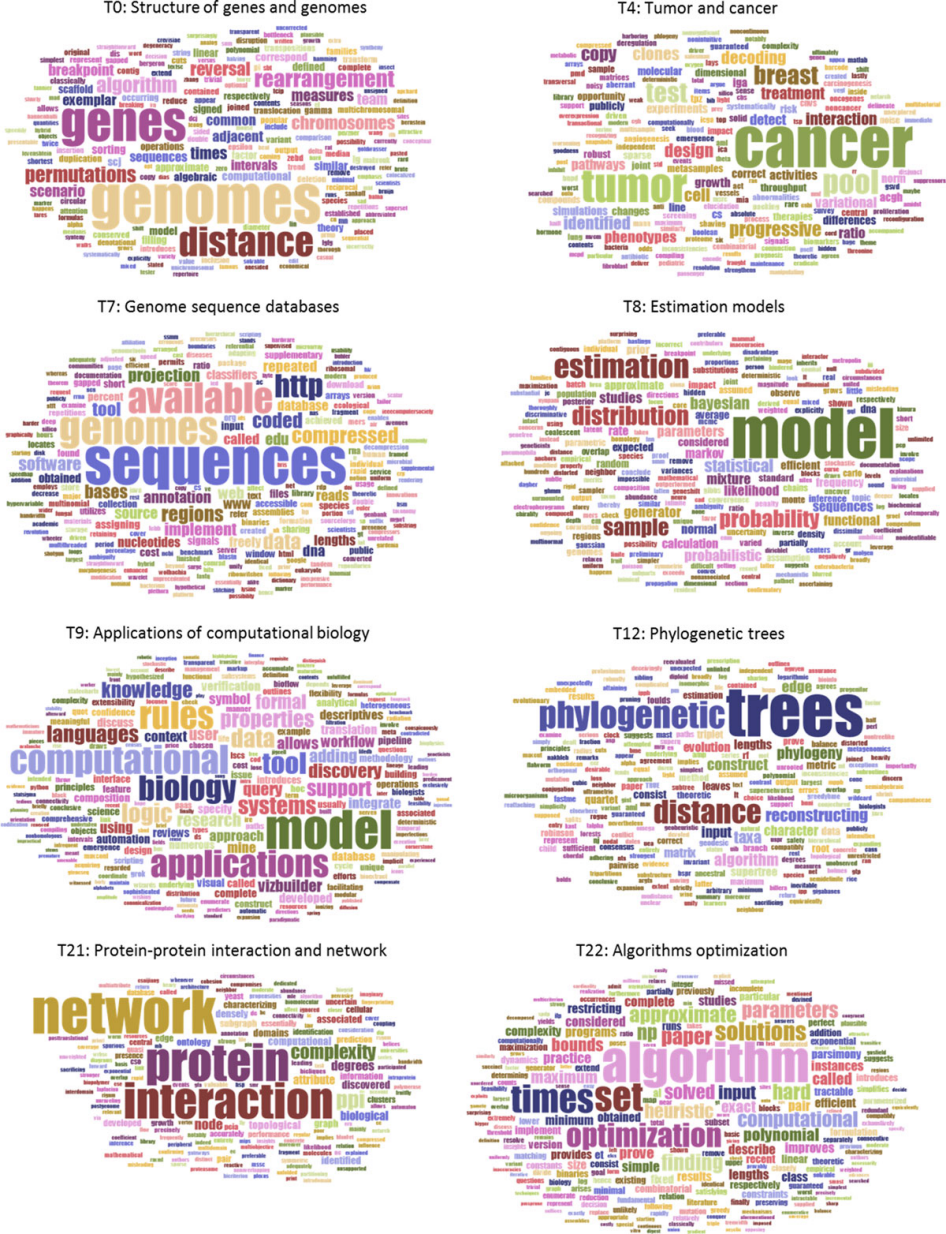
**Eight example topics obtained by LDA modeling with 40 topics on TCBB dataset**.

The TCBB dataset that was downloaded from PubMed database consists of 885 abstracts from ten years of publications in the journal *IEEE Transactions on Computational Biology and Bioinformatics*. Since no truth labels were available to classify them in a manner that would enable a cluster to be built and its purity computed, we used the qualitative approach to assess whether the PRC method could choose the best number of topics. Word clouds were used to represent LDA-derived topic-words matrices, and these matrices were, in turn, subjectively interpreted and evaluated to compare models built with different numbers of topics. Human assessment of topic model validity is a common practice, where topic meaning is subjectively interpreted from the topic-word multinomial distribution. Word clouds are just a way to visualize the distribution where word probabilistic weightings correspond to word graphical (font) sizes. The quality of a model is assessed as higher when its topic themes are more salient and distinguishable than those from other models. The RPC-based method selected 40 as the most appropriate number of topics. We therefore compared the model with 40 topics to the models with 20 and 60 topics. Figure 3 gives word clouds for eight illustrative topics for the model with 40 topics (Suppl. Figure S1 in Additional file 1 ). Each of the eight topic word clouds in Figure 3 depict unique and distinguishable theme, which correspond to distinct research fields of computational biology and bioinformatics. Results (Suppl. Figure S1 in Additional file 1) are similar for the remaining 32 topics. Consider Topic 8 (T8 in Figure 3) for a closer check. Clearly, the salient theme is estimation models, with most words recognizable as pertinent to that field of research. We also located a number of documents in TCBB dataset that had their highest probabilistic association with Topic 8 as listed in Table 5. Most of these papers were, indeed, subjectively judged to be primarily related to estimation models.

**Table 5:** Abstracts with label T8 (Estimation models)

PMID*	Title	Probability of T8
21519119	Inferring the number of contributors to mixed DNA profiles	0.642

21844637	Exploiting the functional and taxonomic structure of genomic data by probabilistic topic modeling	0.568

24384712	Computing the joint distribution of tree shape and tree distance for gene tree inference and recombination detection	0.511

24042552	Computing the Joint Distribution of Tree Shape and Tree Distance for Gene Tree Inference and Recombination Detection	0.474

21030742	The Metropolized Partial Importance Sampling MCMC mixes slowly on minimum reversal rearrangement paths	0.467

21116045	On the distribution of the number of cycles in the breakpoint graph of a random signed permutation	0.398

19407352	Statistical alignment with a sequence evolution model allowing rate heterogeneity along the sequence	0.365

17277422	On the length of the longest exact position match in a random sequence	0.352

20733238	Identifiability of two-tree mixtures for group-based models	0.308

22331862	Faster mass spectrometry-based protein inference: junction trees are more efficient than sampling and marginalization by enumeration	0.291

19179700	The identifiability of covarion models in phylogenetics	0.286

17048396	A short proof that phylogenetic tree reconstruction by maximum likelihood is hard	0.281

18670048	Hadamard conjugation for the Kimura 3ST model: combinatorial proof using path sets	0.267

21233528	Semantics and ambiguity of stochastic RNA family models	0.204

*PMID: PubMed ID number of each paper in Journal of TCBB.

**Figure 4:**
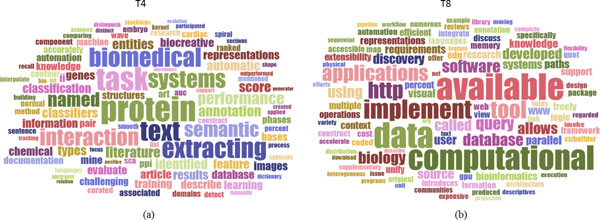
**Two example topics from an LDA model with 20 topics derived from the TCBB dataset**.

**Figure 5:**
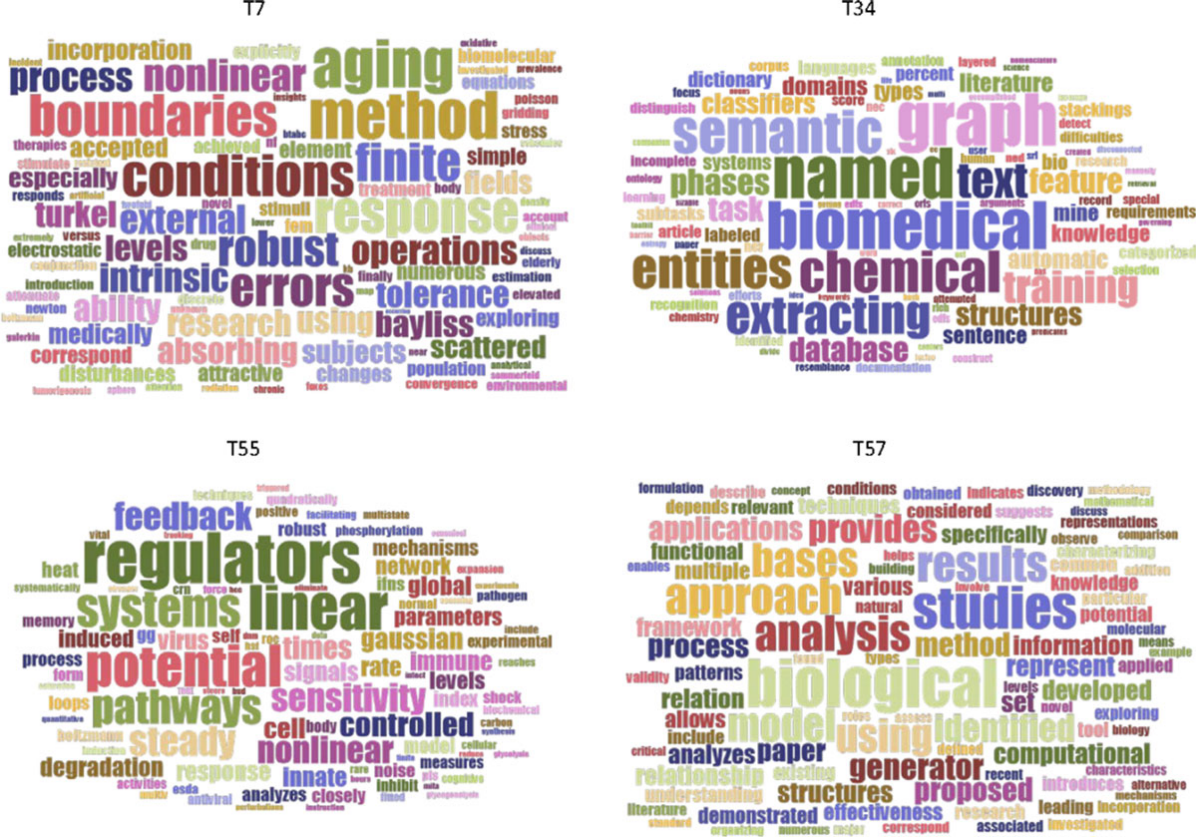
**Four example topics derived by LDA modeling with 60 topics on TCBB dataset**.

For the model with 20 topics, some topics were found salient and distinct themes, and some were not, at least in comparison to the model with 40 topics. Some topics were missing, for example, estimation models such as Topic 8 in Figure 3. Other topics seemed to lump what would preferably be better differentiated themes with 40 topics. For example, the word cloud of T4 shown in Figure 4(a) has at least three themes merged: protein interaction, biomedical task system, and the text extracting. Other topics seemed less specific or too broad as shown in Figure 4(b), compared to those from the model with 40 topics,

In the LDA models with 60 topics, a larger number of topics were judged to be less meaningful in terms of being able to discern a unique and salient theme, compared to the model with 40 topics. Figure 5 gives word cloud representations of four illustrative topics. In each, a few words are displayed with comparable large front size, indicating that these words have comparable high probabilities within the same topic. Consequently, it is hard to distinguish the theme for each topic.

## Discussion

Topic models can often provide highly effective means for text mining and knowledge discovery, especially in the big data era. They are also agnostic as to data type since, for example, biological samples can be considered documents, and gene, protein, biological pathways and many other independent variables can be considered words. There are a myriad of potential applications.

Topic modelling also has drawbacks. They require skill and experience to successfully apply. With all text mining approaches, validation can be difficult, tedious and subjective, where truth is not known *a priori*. Finally, determining the "best model" is an iterative process to determine the parameter values that yield the best outcome, among which is the number of topics.

Currently, a set reasonable guesses or perplexity minimization is mostly used to select an appropriate number of topics for LDA modelling. Both of these approaches are reasonable, but carry a high burden of time and work to carry out the needed sensitivity (parameter) studies. A systematic sensitivity study is further complicated by the variation in models with random seed sampling during the generative model building process.

**Figure 6:**
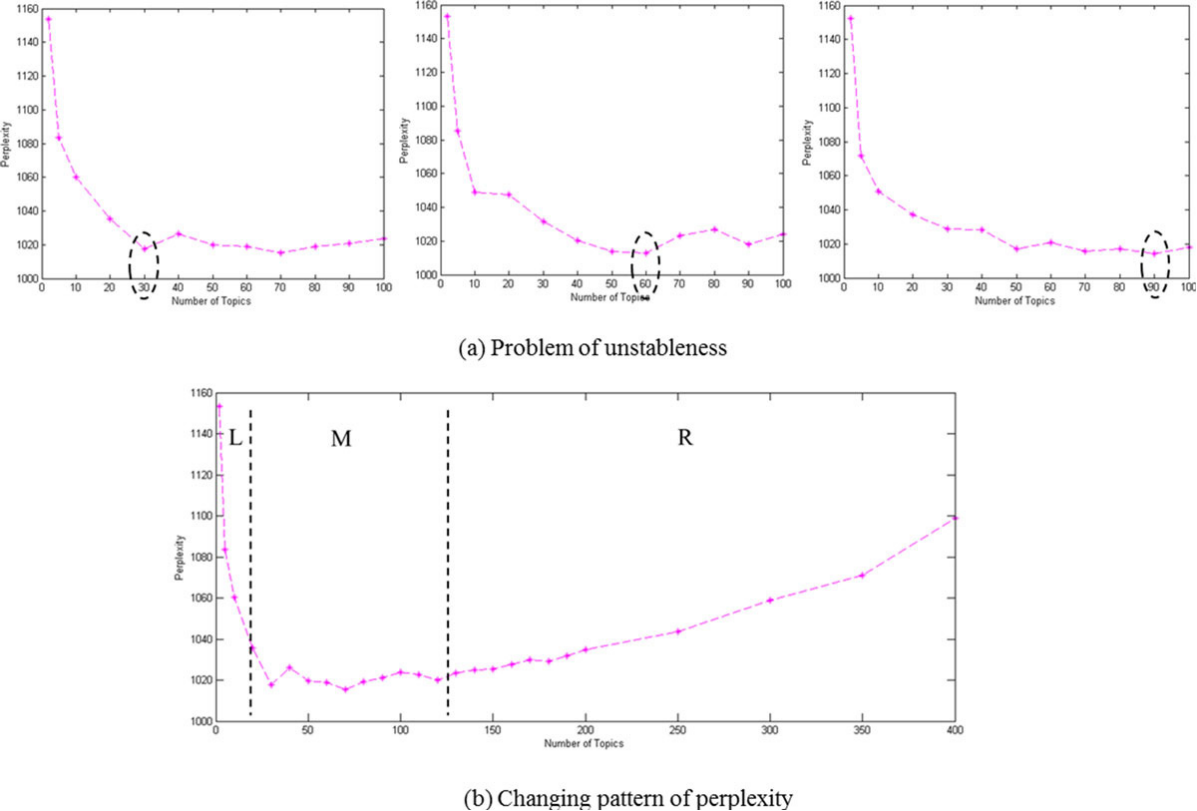
**Two drawbacks of a perplexity-based method in selecting topic numbers**.

Since the objective function in Eq. (1) is a non-convex function, different initial parameters in approximate algorithms, such as Laplace approximation, variational approximation and MCMC, will lead to distinct local maximums. With different random seeds in MCMC or different initial parameters in variational inference approach, the approximate optimizing solutions to LDA may converge to a different local optimal point for the same dataset. As an example, when we applied the perplexity-based method to the *Salmonella *sequence dataset three times with different random seeds in MCMC, very different minimum perplexity values of 30, 60 and 90 (Figure 6(a) were obtained; bear in mind that the leave-one-out cross validation process for each number of topics is carried out with the random seed held constant. Figure 6b shows a plot of perplexity versus number of topics for a wide range of topics up to 400. We can observe the types of variation across number of topics in Figure 6b: (**L**eft section) perplexity decreases steeply as more topics provide a better fit to predict the hold out data; (**M**iddle section) perplexity fluctuates when small variation indicating good fit; and (**R**ight section) perplexity increases due to over fitting of the training set. However, the main concern is that the flattened middle section spans a three-fold range of numbers of topics from 30 to 90, and as shown in Table 1 more than 30 topics results in a much poorer model in terms of accurate serotyping.

The new heuristic approach developed in this study attempts to overcome these weaknesses on the selection of an appropriate number of topics in LDA modelling by offering a heuristic alternative to a full-blown sensitivity study. Rather than choosing among several numbers of topics over a potentially large range where perplexity fluctuates (middle stage M in Figure 6b), the quantity defined as the change-point of rate of perplexity change can be chosen as a putative best number of topics from a heuristic analysis.

We conjecture a theoretical justification for use of RPC-based method on the principle of change-point [18]. For a given series of random variables *x*_1_, *x*_2_,..., *x_T_*, the change-point is distinguished as *t *if a distribution function *F*_1_(*x*) shared by *x*_1_, *x*_2_,..., *x_t _*is different with *F*_2_(*x) *shared by *x_t_, x*_*t*+1_,..., *x_T_*. Applied on the RPC series with increasing candidate topic numbers *T*_1_, *T*_2_,..., *T_K_*, the first number *T_i _*which satisfies *RPC*(*T_i_*) <*RPC*(*T*_*i*+1_) is considered as the most appropriate topic number for the corresponding dataset.

The results confirm that the proposed RPC-based method is stable, accurate and effective for the three numerical experiments presented, each of which constitutes very different data types. In particular, LDA models using numbers of topics from RPC-based selection yielded the matrices for data mining datasets for genomic sequence, drug pharmacology, and textual documents, demonstrating some generalizability across data types. Choosing the best number of topics is an omnipresent concern in topic modelling, as well as other latent variable methodologies. The comparatively simple RPC-based heuristic we propose could simplify topic model development, generally, for many applications, and offer an easier means to use development time for better fine tuning of models.

## Competing interests

The authors declare that they have no competing interests.

## Authors' contributions

WZ (Zhao) performed all the calculations and data analysis, and wrote the first draft of the manuscript. WZ developed the methods, had the original idea, and guided the data analysis and presentation of results. WZ, WZ (Zhao), RP, ZL, YD, and WG participated the dataset construction and result presentation. JC and WZ managed the research and guided the scientific discussing and editing. All authors contributed to data verification, approach evaluation, and assisted with writing the manuscript. All authors read and approved the final manuscript.
